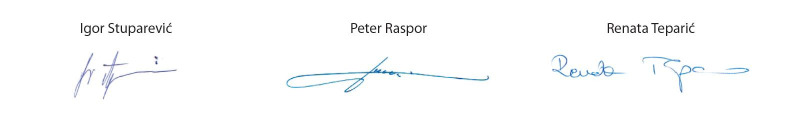# Bridging Yeast Biochemistry and Biotechnological Innovation: A Tribute to Professor Emeritus Vladimir Mrša

**Published:** 2026-02-15

**Authors:** Igor Stuparević, Peter Raspor, Renata Teparić

**Affiliations:** 1 University of Zagreb Faculty of Food Technology and Biotechnology, Pierottijeva 6, Zagreb, Croatia; 2 Emeritus Professor of Microbiology and Biotechnology, University of Ljubljana, Slovenia

This special Issue of *Food Technology and Biotechnology* is dedicated to Professor Emeritus Vladimir Mrša, a long-time editor of the journal whose expertise and guidance have greatly influenced biotechnology in Croatia. For over four decades, his research advanced our understanding of yeast-secreted mannoproteins and the complex mechanisms of cell wall biogenesis. He made significant contributions to the global understanding of yeast cell wall glycoprotein functions and pioneered the development of yeast cell wall display systems for immobilising recombinant proteins, a technique with immense potential for modern biotechnological processes. Vladimir Mrša is remembered not only for his sharp intellect but also for the genuine warmth and mentorship he offered to colleagues and students. This issue includes both original research and review articles offering novel insights expected to greatly benefit the bioscience field. To mark his contributions in shaping the landscape of yeast research and his editorial activities, we have compiled this special issue with a selection of papers that reflect his broad perspective on the biosciences.

Among review articles, T. Martinić Cezar *et al.* explore enhancing yeast surface display by reviewing the endoplasmic reticulum (ER) as a crucial bottleneck for protein immobilization. They focus on how the unfolded protein response (UPR) and ER-associated degradation (ERAD) affect production efficiency. The secretory pathway acts as a filter, allowing more stable protein mutants to fold easily and achieve higher copy numbers on the surface.

Z. Montazer and K. Khosravi-Darani explore dual-target bioprocessing using oleaginous microorganisms to transform food waste into valuable products such as single-cell oils (SCOs) and polyhydroxyalkanoates (PHAs). This innovative method reduces production costs and supports a circular bioeconomy by promoting sustainable alternatives to fossil fuel-based resources. By directly assimilating volatile fatty acids from fruit and vegetable residues, the process eliminates the need for costly enzymatic hydrolysis, reducing operational costs. Successful simultaneous production of SCOs and PHAs depends on carefully controlling the carbon-to-nitrogen ratio to prevent interference between the metabolic pathways involved.

T. Lukman and S. Smole Možina review monofloral bee pollen and the impact of botanical origin on its antimicrobial potential. They emphasize that processing methods such as enzymatic hydrolysis and fermentation disrupt the resistant pollen wall (exine) to enhance the bioavailability of bioactive compounds. Fragmenting the tough outer pollen wall through mechanical shear or ultrasonication is essential for making internal nutrients bioavailable for human consumption. Comparative studies show that lyophilization is the most effective stabilization method for preserving the biological potency of various botanical species.

M. Jevšnik Podlesnik and P. Raspor examine the critical role of human behaviour and organizational culture in ensuring food safety. They argue that true safety requires moving beyond regulatory compliance toward a transformation that internalizes food safety values. Different nudge tools, such as specific scents or visual cues, have proven to be subtle yet effective tools for increasing hygiene compliance in professional settings. The integration of artificial intelligence into food management systems offers new opportunities to support human decision-making while reducing repetitive workloads.

S. Schmid *et al.* review the recombinant production of vanillin using hosts such as bacteria, fungi and microalgae, emphasizing the legislative standard for ‘natural’ vanillin. Their work addresses the challenge of vanillin toxicity to host cells and discusses *in situ* product removal to achieve industrially relevant yields. Using glucose as a primary substrate is highly economical and preferred, as it is non-toxic to microorganisms compared to phenolic precursors. Metabolic engineering efforts often involve knocking out specific alcohol dehydrogenases to prevent the undesired reduction of vanillin to vanillyl alcohol.

Among research papers, K. Butorac *et al.* evaluated the potential of strains of lactic acid bacteria isolated from human milk as functional starter cultures. They identified specific strains with high proteolytic, antimicrobial and antioxidant activities, ensuring safety and nutritional relevance. Incorporating these proteinase-active strains into infant formulas could significantly improve casein digestibility for newborns. The presence of S-layer proteins on the cell surface of certain *Levilactobacillus brevis* strains provided critical protection during freeze-drying.

Functional characterization of the Gmh5p protein by M. Lommel *et al.* demonstrates that it is a mannosyltransferase homologous to *Saccharomyces cerevisiae* Mnn10p. This enzyme is involved in N-glycan outer chain elongation, essential for maintaining cell wall integrity. Restoring tolerance to the antibiotic hygromycin B in *S. cerevisiae* mutants demonstrated the functional conservation of this enzyme across distantly related species. This research corrects previous bioinformatic models that inaccurately predicted the enzyme’s function as a galactosyltransferase.

M. Mrkonjić Fuka *et al.* investigated the antibacterial activity of royal jelly against multidrug-resistant (MDR) pathogens, including MRSA and VRE. They found that royal jelly inhibited MDR bacteria with efficacy varying at the strain level, suggesting synergistic effects from its complex composition. Chemical profiling identified octanoic acid as the dominant volatile compound, likely playing a major role in the antimicrobial properties of the substance. Susceptibility testing showed that while *Acinetobacter baumannii* was easily inhibited, *Enterococcus faecium* strains remained the most resistant to treatment.

M. Avbelj *et al.* used targeted genome engineering to achieve oxytetracycline hyper-production in the antibiotic-producing organism. By introducing large deletions, they showed that even ‘silent’ biosynthetic gene clusters can be activated to affect metabolism. Targeted removal of a 145 kb chromosomal region led to a 50-fold upregulation of the specific genes responsible for antibiotic production. Surprisingly, deleting an entirely silent gene cluster increased antibiotic yields by over 70 %, suggesting complex interactions within the genome.

A single-tube loop-mediated isothermal amplification (LAMP) assay targeting the *inlA* gene was developed by A. Maraz *et al.* for rapid detection of *L. monocytogenes*. This method combines DNA extraction and colorimetric sensing, making it suitable for integration into microfluidic platforms. DNA extraction was efficiently achieved using a Triton X-100-based buffer, which significantly outperformed alternative alkaline lysis methods. Testing confirmed that the assay is highly reliable, achieving a sensitivity of 100 % and a specificity of 96 % when targeting the *inlA* virulence gene.

Professor Mrša’s transformative leadership as Editor-in-Chief since 2009 elevated *Food Technology and Biotechnology* to international prominence, as he steadfastly advocated the principles of diamond open access and scholarly excellence. His enduring legacy lives on through his pioneering research in yeast biochemistry and cell wall display systems, as well as in the careers of the many colleagues and students he inspired with his selfless mentorship and genuine warmth. In addition to his work on yeast, Vladimir Mrša made lasting contributions to education, research and international cooperation in the fields of molecular biology and biotechnology. Professor Mrša consistently welcomed new methodologies and interdisciplinary approaches, readily integrating emerging molecular techniques into his laboratory’s work. He was known for encouraging young researchers to explore novel ideas and for building bridges with international partners, creating a collaborative environment where scientific curiosity and cooperation thrived. This special issue includes articles from scientists in various countries with whom professor Mrša collaborated on research topics, as well as in his roles as a journal editor and organiser of conferences. Although his passing leaves a significant void in the scientific community, we dedicate this special Issue to his memory, honouring a remarkable scientist whose vision and friendship will continue to guide and inspire us for generations to come.